# Targeting *HSF1* sensitizes cancer cells to HSP90 inhibition

**DOI:** 10.18632/oncotarget.991

**Published:** 2013-04-23

**Authors:** Yaoyu Chen, Jinyun Chen, Alice Loo, Savina Jaeger, Linda Bagdasarian, Jianjun Yu, Franklin Chung, Joshua Korn, David Ruddy, Ribo Guo, Margaret E. Mclaughlin, Fei Feng, Ping Zhu, Frank Stegmeier, Raymond Pagliarini, Dale Porter, Wenlai Zhou

**Affiliations:** ^1^ Oncology, Novartis Institutes for Biomedical Research, Cambridge, MA, USA; ^2^ Oncology Translational Research, Novartis Institutes for Biomedical Research, Cambridge, MA, USA; ^3^ Oncology, Novartis Institutes for Biomedical Research, Emeryville, CA, USA

**Keywords:** HSF1, cancer cells, HSP90 inhibitor, Melanoma, HCC, DEDD2

## Abstract

The molecular chaperone heat shock protein 90 (HSP90) facilitates the appropriate folding of various oncogenic proteins and is necessary for the survival of some cancer cells. HSP90 is therefore an attractive drug target, but the efficacy of HSP90 inhibitor may be limited by HSP90 inhibition induced feedback mechanisms. Through pooled RNA interference screens, we identified that heat shock factor 1(*HSF1*) is a sensitizer of HSP90 inhibitor. A striking combinational effect was observed when *HSF1* knockdown plus with HSP90 inhibitors treatment in various cancer cell lines and tumor mouse models. Interestingly, *HSF1* is highly expressed in hepatocellular carcinoma (HCC) patient samples and HCC is sensitive to combinational treatment, indicating a potential indication for the combinational treatment. To understand the mechanism of the combinational effect, we identified that a *HSF1*-target gene *DEDD2* is involved in attenuating the effect of HSP90 inhibitors. Thus, the transcriptional activities of *HSF1* induced by HSP90 inhibitors provide a feedback mechanism of limiting the HSP90 inhibitor's activity, and targeting *HSF1* may provide a new avenue to enhance HSP90 inhibitors activity in human cancers.

## INTRODUCTION

Molecular chaperones assist in the folding of nascent polypeptides and the correct assembly or disassembly of protein complexes [1, 2]. A majority of chaperones are the so-called heat-shock proteins (HSPs), which are expressed in response to increased temperature or a variety of other cellular stresses. Among them, heat shock protein 90 (HSP90) is a conserved molecular chaperone and is involved in stabilizing and activating more than 200 proteins[2]. Since many HSP90 ‘clients' are known oncogenic proteins, such as tyrosine kinases[3-5], steroid hormone receptors[6], AKT[7], HIF1α[8] and MMP2[9], that are known to sustain cancer cell growth, differentiation and survival. HSP90 chaperone machinery enables mutated oncoproteins to escape from misfolding and degradation and allows for malignant transformation [2, 10, 11]. Therefore, HSP90 is considered as a synthetic lethal target [12-14].

After the first HSP90 inhibitor, 17-AAG (tanespimycin), entered clinical trials in 1999, thirteen different HSP90 inhibitors are currently undergoing clinical evaluation in cancer patients in twenty-three active oncology trials [15]. Each of these inhibitors disrupts HSP90 activity by replacing ATP in the N-terminal nucleotide-binding pocket [11, 15]. NVP-AUY922 and NVP-HSP990 are novel, non-geldanamycin-derivative HSP90 inhibitors [16]. Both compounds showed significant antitumor activity in a wide range of mutated and wild-type cancer cell lines, primary tumor cells and animal models of cancer, including melanoma, myeloma, gastric cancer, non-small-cell lung cancer(NSCLC), hepatocellular cancer, sarcoma, and breast cancer [16-19]. Progress has also been made in terms of identifying sensitive cancer indications and effective drug combinations: in HER2+ breast cancer, HSP90 inhibitors block HER2 signaling and suppress tumor growth as the stability of HER2 protein is dependent on HSP90. In a Phase II clinical trial following combination of trastuzumab with 17-AAG treatment, a response rate of 24% was reported and clinical benefit was observed in more than 57% of evaluated patients[2, 20].

Although significant progress has been made and promising results have been seen in breast cancer patients receiving HSP90 inhibitor treatment, HSP90 inhibitor was also shown to lack efficacy in certain cancer types, such as melanoma. In a Phase II trial of 17-AAG in patients with metastatic melanoma, no objective anti-melanoma responses were observed [21]. Therefore, understanding the resistant mechanisms of cancer cells in response to HSP90 inhibition will help us to develop the effective combinational therapy with HSP90 inhibitor.

To identify the genetic modulators of HSP90 inhibition, we performed pooled shRNA screening to search the potential combinational targets of HSP90 inhibitor, and identified *HSF1* as a sensitizer of HSP90 inhibitor. HSF1 is a conserved transcription factor and a major regulator of the heat shock response [22, 23]. Beyond heat shock response, HSF1 also regulates a transcriptional program highly specific to malignant cell including cell cycle, cell signaling, metabolism, adhesion and translation [23-25]. Recently, eliminating HSF1 was showed to protect mice from tumors induced by mutation of the RAS oncogene or a hot spot mutation in tumor suppressor p53 and from DEN-induced hepatocellular carcinoma (HCC) formation [24, 26]. Loss of tumor suppressor NF1 activates HSF1 to promote carcinogenesis through dysregulated MAPK signaling [27]. Moreover, HSF1 knock-out or knock-down cells were shown to be more sensitive to HSP90 inhibitor [28-31]. Those studies indicate that HSF1 may play an important role in tumor initiation, development and maintenance, and contribute to cell sensitivity to HSP90 inhibitor. However, the functional role of HSF1 in human cancer cell resistance to HSP90 inhibitors and the mechanisms underlying the combination effect of *HSF1* knockdown and HSP90 inhibitors are not fully understood. Moreover, the downstream targets of HSF1 which may play a role in attenuating the effect of HSP90 inhibitor are not fully appreciated.

In this study, we observed that *HSF1* knockdown combined with HSP90 inhibitors led to striking inhibitory effects on cancer cell proliferation *in vitro* and tumor growth *in vivo*. *HSF1* knockdown combined with HSP90 inhibition facilitates the degradation of oncogenic proteins, induces cancer cell apoptosis, and decreases activity of the ERK pathway. HSF1 expression is significantly up-regulated in HCC, suggesting a tumor type that may be targeted by combinational treatment. Finally, we identify *DEDD2* as a HSF1 target gene involved in the resistance to HSP90 inhibition.

## RESULTS

### Pooled shRNA screening reveals that *HSF1* as a top sensitizer to HSP90 inhibitor

**Figure 1:**
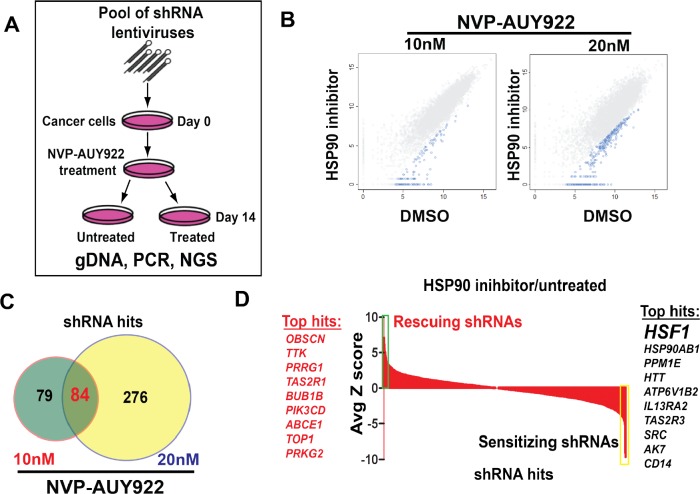
Pooled shRNA screening reveals that *HSF1* as a top sensitizer to HSP90 inhibitor A. The schematic of pooled shRNA screening experiment design. B. Scatter plots of log2 normalized read counts from pooled shRNA screening performed in A375 cells treated with 10nM/20nM NVP-AUY922 or control dimethyl sulfoxide (DMSO) samples. Hairpins that were statistically significantly depleted in NVP-AUY922 treated samples were highlighted in blue color. Each dot in the plot represents one individual shRNA construct. C. Venn diagram showed that 163 shRNAs were identified in lower dose NVP-AUY922 and 360 shRNAs were identified from higher dose NVP-AUY922 from pooled shRNA screening performed in A375 cell. 84 shared shRNAs were found between two experiments. D. Average Z score of shRNA hits from pooled shRNA screening performed in A375 cells was shown in a waterfall plot. Top sensitizing genes including *HSF1* were highlighted in yellow color while top rescuing genes were shown in green.

To identify genes that modulate the efficacy of HSP90 inhibition on tumor cell growth, we performed a large-scale RNA interference (RNAi) genetic screen with a collection of short hairpin RNA (shRNA) vectors targeting 1,000 human genes in A375 (Fig. 1A). A barcoding technique was used to identify genes whose suppression caused resistance or sensitivity to two separate concentrations of NVP-AUY922 (Fig. 1B). 163 and 360 shRNA constructs were significantly depleted form either low- or high-dose NVP-AUY922 treated samples (FDR<=0.15). Among those shRNA hits, 84 hits (including 81 genes) were common shRNA hits as shown in Venn diagram (Fig. 1C) and sensitizing genes or rescuing genes were also shown (Z score≥3, or Z score≤-3, Supplementary Table. S1). Among of these shRNA hits, *HSF1* and heat shock protein 90 alpha, class B member 1(*HSP90AB1*) knockdown scored as the most top sensitizers to HSP90 inhibition in A375 cells, and are known to regulate the cell response to heat shock conditions (Fig. 1D), which may reflect the potential feedback mechanism of HSP90 inhibition. Taking together, HSF1 is identified as a sensitizer of HSP90 inhibitor through pooled shRNA screening.

### *HSF1* knockdown sensitizes cancer cells to HSP90 inhibitor in vitro and *in vivo*

**Figure 2:**
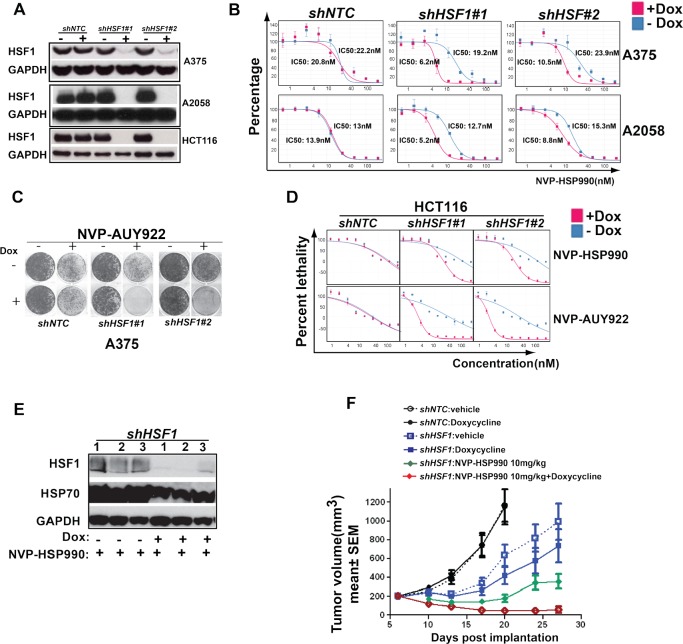
*HSF1* knockdown sensitizes cancer cells to HSP90 inhibitor *in vitro* and *in vivo* A. Western blotting analysis of HSF1 knockdown in A375, A2058 and HCT116 cells. *shNTC* or *shHSF1* transduced stable cell lines were treated with Doxycycline for 3 days and cell pellets were collected and western blotting was performed. B. IC50 of HSP90 inhibitor with or without *HSF1* knockdown. *shNTC* or *shHSF1* transduced cancer cells were treated with or without Doxycycline for 3 days, then followed by treatment of a serial dilutions of NVP-HSP990 for 5 days. Relative cell growth (average of at least 3 independent experiments) was measured by CellTiter-Glo and normalized to DMSO-treated cells. C. Cell colony formation assay of *HSF1* knockdown with HSP90 inhibitor treatment. *shNTC* or *shHSF1* transduced A375 cells were treated or untreated with Doxycycline for 5 days, then followed by compound treatment for 6 days. D. LD50s of HSP90 inhibitors: NVP-AUY922 and NVP-HSP990 with or without *HSF1* knockdown in HCT116 cells. *shNTC* or *shHSF1* transduced cancer cells were treated with or without Doxycycline for 3 days, then followed by being treated for 5 days with serial dilutions of NVP-HSP990 or NVP-AUY922. Relative cell growth (average of at least 3 independent experiments) was measured by CellTiter-Glo. Cell relative death rate was calculated. E. Western blotting analysis of tumor samples. Tumor samples were collected at the end of studies and western blotting analysis of HSF1, HSP70 and GAPDH were performed. F. The combinational effect of *HSF1* knockdown and HSP90 inhibitor in A375 xenograft mouse model. Tumor growth rate of A375 cells expressing inducible control shRNA or shRNA against *HSF1* under Doxycycline and/or NVP-HSP990 were compared at different time points. Tumor inhibition effects were calculated relative to control group at day 20.

To validate whether *HSF1* was indeed a sensitizer of HSP90 inhibition, two *HSF1* inducible shRNA constructs by targeting distinct *HSF1* sequence were stably introduced into different cancer cell lines: A375, A2058 and HCT116. When shRNA expression was induced by Doxycycline, robust *HSF1* knockdown was achieved in all three cancer cell lines (Fig. 2A). We next tested whether *HSF1* knockdown has a combinational effect with NVP-AUY922 or NVP-HSP990. Induction of *HSF1* shRNA (but not the NTC shRNA) led to IC50 of NVP-HSP990 shifting from 19nM to 6nM in A375 cell, 12.7nM to 5.2nM in A2058 cell (Fig. 2B). The combination effect was even more dramatically observed in extended colony formation assays (Fig. 2C). In HCT116 cells, *HSF1* knockdown led to a significant shift of LD50 of either NVP-HSP990 or NVP-AUY922 (more than 6 fold change) (Fig. 2D). To further validate *HSF1* as a sensitizer of HSP90 inhibitor, the combinational effect of *HSF1* knockdown with HSP90 inhibitor was tested in A375 xenograft mouse model. *HSF1* shRNA alone inhibited tumor growth by 53% T/C, and knockdown was confirmed (Fig. 2E and F). NVP-HSP990 alone at tolerated dosage (10mg/kg PO, qw) inhibited tumor growth by 0.01% T/C (Fig. 2F). More strikingly, *HSF1* knockdown & NVP-HSP990 combination led to 76% tumor regression (Fig. 2F). HSP70 level induced by HSP90 inhibition was also significantly reduced upon *HSF1* knockdown (Fig. 2E). These results suggest that HSF1 is critical for limiting the efficacy of HSP90 inhibitor in human cancer cells both in *vitro* and in *vivo*.

### *HSF1* knockdown sensitizes HCC cells to HSP90 inhibition

**Figure 3:**
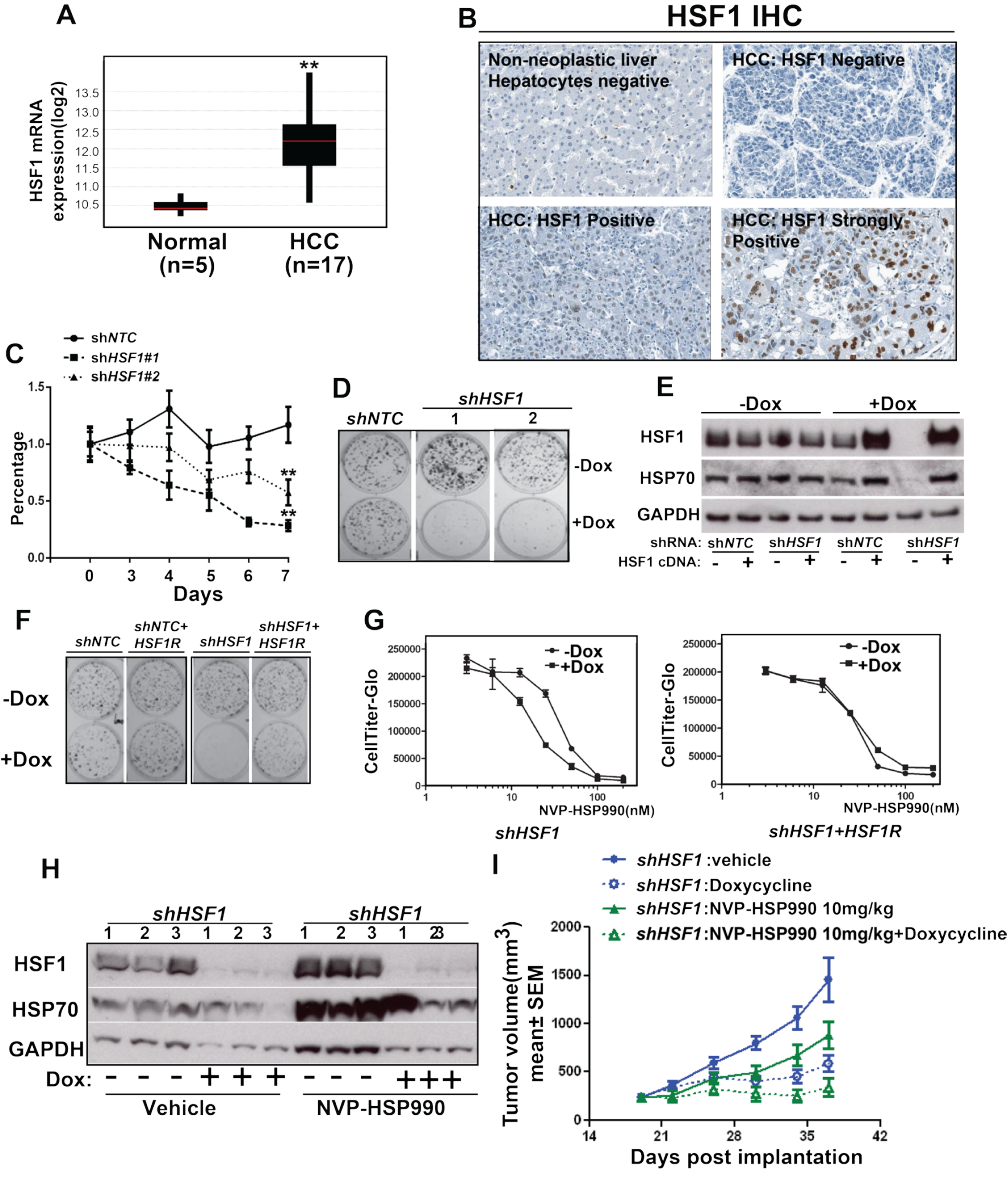
HSF1 knockdown sensitizes hepatocellular cancer cells to HSP90 inhibitor *in vitro* and *in vivo* A. The mRNA level of *HSF1* was measured among tumor samples from HCC patients compared to normal control. B. IHC showed the expression of HSF1 in primary human hepatocellular cancer. C. The cell growth curve of Hep3B cell with or without *HSF1* knockdown at different time points. *shNTC* or *shHSF1* transduced Hep3B cells were treated with or without Doxycycline for 7 days. Relative cell growth (average of at least 3 independent experiments) was measured by CellTiter-Glo and normalized to cells without Doxycycline treatment. D. Cell colony formation assay of *HSF1* knockdown in Hep3B cells. *shNTC* or *shHSF1* transduced Hep3B cells were treated with Doxycycline for 12 days. E. Western blotting analysis of Hep3B cells expressing the indicated shRNA and cDNA contruct. The inducible lenti-virus construct (*HSF1*R cDNA) was used to transduce Hep3B cells and expression of *HSF1R* cDNA with or without *HSF1* knockdown were tested. HSF1, HSP70 and GAPDH were detected by western blotting. F. Cell colony formation assay of over-expression of *HSF1*R cDNA in Hep3B cells with or without *HSF1* knockdown. G. The comparison of dose response of NVP-HSP990 in Hep3B cells with inducible expression of either *HSF1* shRNA alone or both *HSF1* shRNA and *HSF1R* cDNA. *HSF1* shRNA alone or both *HSF1* shRNA and *HSF1R* cDNA transduced cancer cells were treated with or without Doxycycline for 3 days, then followed by being treated for 5 days with serial dilutions of NVP-HSP990. H. Western blotting analysis of tumor samples. Tumor samples were collected at the end of studies and western blotting analysis of HSF1, HSP70 and GAPDH were performed. I. The combinational effect of *HSF1* knockdown and HSP90 inhibitor in Hep3B xenograft mouse model. Tumor growth rate of Hep3B cells expressing inducible control shRNA or shRNA against *HSF1* under Doxycycline and/or NVP-HSP990 were compared at different time points.

To identify the cancer type which may be useful for the stratification of the combinational treatment, we examined HSF1 expression in TCGA database and found that *HSF1* is over-expressed in several tumor types (Supplementary Figure S1). Among them, we are particularly interested in HCC since HSF1 has been reported to be a key modulator of HCC development in mouse model [26]. *HSF1* mRNA was significantly elevated in HCC tumor in compared to normal control (Fig. 3A). To examine the protein expression of HSF1 in HCC patients, immunohistochemistry (IHC) study for HSF1 was performed on primary human HCC samples and non-neoplastic liver samples and the HSF1 antibody was validated by using Hep3B cell pellets with inducible *HSF1* shRNAs. HSF1 staining was significantly decreased in Hep3B cells treated with Doxycycline in comparison with cells without Doxycycline treatment (Supplementary Fig. S2). HSF1 staining was then scored in 50 human HCC tumor samples. Samples that were not immunoreactive (Ki67 negative) or had insufficient tumor were excluded from the analysis. HSF1 expression was not observed in non-neoplastic human hepatocytes of the 45 HCC tumor samples were evaluable, 35 HCC cases showed positive HSF1 staining (Fig. 3B and Table 2). The intensity of staining varied considerably from tumor to tumor. These results suggest that both mRNA and protein level of HSF1 expression are increased in HCC.

Next, we tested whether *HSF1* knockdown could inhibit the proliferation of HCC and whether there is a combinational effect of *HSF1* knockdown and HSP90 inhibitor in HCC cells. Indeed, both the cell proliferation and colony formation assay showed that *HSF1* knockdown inhibited the growth of HCC *in vitro* (Fig. 3C and D). In addition, *HSF1* knockdown sensitized both Hep3B and Huh7 cells to HSP90 inhibitor at different levels *in vitro* (Table 1). To evaluate whether the phenotype mediated by *HSF1* shRNA knockdown and the combination effect mediated by *HSF1* shRNA knockdown and HSP90 inhibition was on-target, RNAi-resistant *HSF1* (“HSF1R”) cDNA was expressed in Hep3B cells with inducible *HSF1* knockdown. *HSF1*R cDNA restored the expression of HSF1 and HSP70 in Hep3B cell lines when endogenous HSF1 and HSP70 expression was decreased by Doxycycline treatment (Fig. 3E). *HSF1*R cDNA expression rescued both growth phenotype medicated by *HSF1* knockdown (Fig. 3F) and the combinational effects caused by HSF1 knockdown and HSP90 inhibition (Fig. 3G), which indicate that the effects of the *HSF1* shRNA constructs are indeed on-target effects. The effect of *HSF1* knockdown on the proliferation of tumor cells was also tested in Hep3B xenograft mouse model. *HSF1* shRNA alone inhibited tumor growth by 71%, and knockdown was confirmed (Fig. 3H) and NVP-HSP990 alone at tolerated dosage (10mg/kg PO, qw) inhibited tumor growth by 47% (Fig. 3I). However, *HSF1* knockdown & NVP-HSP990 combination reduced the HSP90 inhibitor induced cell stress response and led to tumor stasis (Fig. 3H and I).

**Table 1:** *HSF1* knockdown sensitizes cancer cells to HSP90 inhibitor The combinational effect of *HSF1* knockdown and HSP90 inhibition was observed among 2 melanoma cell lines, 2 HCC lines and 1 colon cancer cell line

	*In vitro*		*In vivo*
Cell line	IC50	LD50	
A375	2.9	N/A	Regression
A2058	2	N/A	
HCT116	N/A	6.5	
Hep3B	2.1	1.9	Stasis
Huh7	3.1	7	

Average fold change of IC50 or LD50 of HSP90 inhibitor caused by HSF1 knockdown

**Table 2:** Immunohistochemistry study showed that the expression of HSF1 is significantly up-regulated in primary human hepatocellular cancer The expression of HSF1 was measured by immunohistochemistry (IHC) in hepatocellular carcinomas (50 Hep B virus positive cases, 2 cores/case).

Hepatocellular carcinoma			
Case coordinates	HSF1	Ki67	IgG
A1,2	POS-very weak	POS	NEG
A3,4	POS	POS	NEG
A5,6	POS-strong	TI	NEG
A7,8	POS	TI	NEG
B1,2	TI	TI	TI
B3,4	POS-strong	POS	NEG
B5,6	POS	POS	NEG
B7,8	POS-weak	POS	NEG
B9,10	POS-very weak	TI	NEG
B11,12	NEG	TI	NEG
C1,2	POS	POS	NEG
C3,4	NEG	POS	NEG
C5,6	NEG	POS	NEG
C7,8	POS	POS	NEG
C9,10	POS	POS	NEG
C11,12	POS	POS	NEG
D1,2	POS	POS	NEG
D3,4	NEG	POS	Brown pigment c/w lipofuscin
D5,6	POS	POS	NEG
D7,8	NEG	POS	NEG
D9,10	POS	POS	NEG
D11,12	POS-weak	POS	NEG
E1,2	NEG	POS	NEG
E3,4	POS-very weak	POS	NEG
E5,6	NEG	POS	Brown pigment c/w bile
E7,8	POS	POS	NEG
E9,10	POS-strong	POS	NEG
E11,12	POS-weak	POS	NEG
F1,2	POS-weak	POS	NEG
F3,4	POS-weak	POS	NEG
F5,6	NEG	POS	NEG
F7,8	POS-very weak	POS	NEG
F9,10	NEG	POS	NEG
F11,12	POS-weak	POS	NEG
G1,2	POS	POS	NEG
G3,4		NEG	NEG
G5,6		NEG	NEG
G7,8	POS-weak	POS	NEG
G9,10	POS-weak	POS	NEG
G11,12	POS-weak	POS	NEG
H1,2	POS-very weak	POS-very weak	NEG
H3,4	POS	POS	Minimal brown pigment
H5,6	POS-very weak	POS	NEG
H7,8	POS-very weak	POS-very weak	NEG
H9,10	POS-very weak	POS	NEG
H11,12	POS	POS	NEG
I1,2		NEG	NEG
I3,4	POS-very weak	POS	NEG
I5,6	NEG	POS	NEG
I7,8	NEG	POS	NEG
A9,10 (non-neoplastic)	Hepatocytes negative; inflammatory cells positive	TI	NEG
A11,12 (non-neoplastic)	Hepatocytes negative; inflammatory cells positive	TI	Brown pigment c/w lipofuscin

### Combination of *HSF1* knockdown and HSP90 inhibition leads to a decreased level of p-ERK and an increase of cell apoptosis

**Figure 4:**
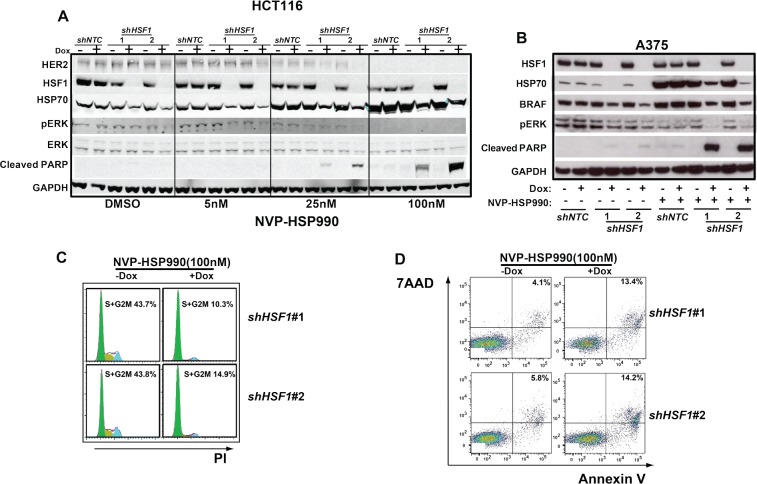
Combination of *HSF1* knockdown and HSP90 inhibitor leads to a decreased level of p-ERK and an increased cell apoptosis rate A. Western blotting analysis of HCT116 cells expressing the inducible sh*HSF1* treated with different doses of NVP-HSP990. *shNTC* or *shHSF1* transduced HCT116 cells were treated with or without Doxycycline for 3 days and were further treated with different doses of NVP-HSP990 for 48h. B. Western blotting analysis of A375 cells expressing the inducible shRNA treated with different doses of NVP-HSP990. *shNTC* or *shHSF1* transduced A375 cells were treated with or without Doxycycline for 3 days and were further treated with NVP-HSP990 100nM for 48h. C. Cell cycle analysis of A375 cells treated with NVP-HSP990 with or without *HSF1* knockdown. *shHSF1* transduced A375 cells were treated with or without Doxycycline for 3 days and were further treated with NVP-HSP990 100nM for 48h. The percentage of S+G2M cells were determined by PI staining. D. Cell apoptosis analysis of A375 cells with *HSF1* knockdown and NVP-HSP990 treatment. *shHSF1* transduced A375 cells were treated with or without Doxycycline for 3 days and were further treated with NVP-HSP990 100nM for 48h. The apoptotic cells represented by 7AAD+AnnexinV+ were determined by FACS.

To understand the mechanism of the combination effects of *HSF1* knockdown and HSP90 inhibition, we tested: 1) whether *HSF1* knockdown may facilitate the degradation of HSP90 client protein by HSP90 inhibition, such as BRAF or HER2 oncogenic proteins; 2) whether HSP90 inhibition may enhance the attenuation of MAPK signaling mediated by HSF1 knockdown as recent finding suggests that *HSF1* deficiency attenuates MAPK signaling in mice[27] and 3) HSF1 may regulate other target genes rather than HSP70, which may play a role in attenuating the effect of HSP90 inhibition. Therefore, we examined the status of HSP90 client proteins and the downstream effects in cell treated with either *HSF1* shRNA or HSP90 inhibitor or combination of HSF1 shRNA and HSP90 inhibitor. HCT116 cells were treated with different doses of NVP-HSP990 and HSF1 knockdown in combination with NVP-HSP990 (5nM) reduced the HSP70, p-ERK and HER2 levels significantly while NVP-HSP990 or *HSF1* knockdown alone did not(Fig. 4A). HSF1 knockdown in combination with NVP-HSP990 (25nM) led increased cleaved PARP (Fig. 4A). The combination also led an enhanced degradation of BRAF in A375 cells (Fig. 4B). A decreased level of HSP70, p-ERK and increased level of cleaved PARP were also observed in melanoma cells (Fig. 4B) and hepatocellular cancer cells (Supplementary Fig. S3). To understand how *HSF1* knockdown affects the cell proliferation under HSP90 inhibitor treatment, cell cycle analysis was performed. *HSF1* knockdown didn't affect the percentage of cancer cells in cell cycle while HSP90 inhibitor caused more cancer cells into S+G2M phase (data not shown). In contrast, the percentage of cancer cells in the S+G2M phase was significantly decreased in *HSF1* knockdown group than in the control group under HSP90 inhibitor treatment(Fig. 4C), indicating that under HSP90 inhibition the knockdown of *HSF1* blocks cancer cells to enter the cell cycle, thereby decrease the proliferation of cancer cells. Next, we examined whether *HSF1* knockdown may enhance apoptosis of cancer cells under HSP90 inhibitor treatment by staining the cells with 7AAD and Annexin V. Similarly, *HSF1* knockdown didn't affect the apoptosis of cancer cells while HSP90 inhibitor induced the apoptosis of cancer cells (data not shown). *HSF1* knockdown further enhanced the apoptotic proportion of cancer cells under HSP90 inhibitor treatment (Fig. 4D). Thus, the combination treatment of *HSF1* knockdown and HSP90 inhibition facilitates the degradation of HSP90 client proteins, such as BRAF and HER2, inhibits MAPK growth signaling and results in cell cycles arrest and cell apoptosis.

### *DEDD2* is a HSF1-target gene involved in attenuation of the effect of HSP90 inhibitor

**Figure 5:**
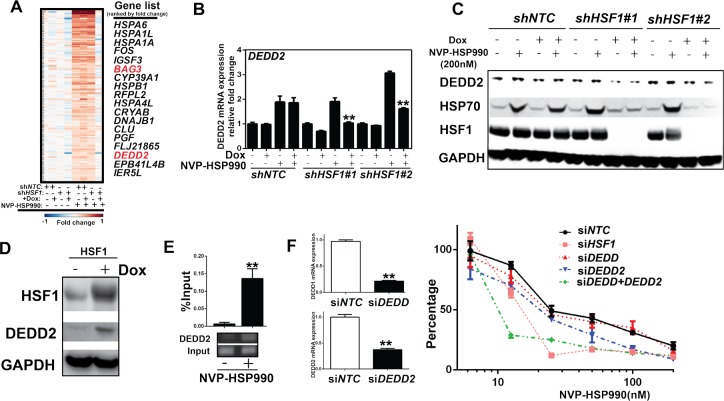
*DEDD2* is a HSF1-target gene involved in attenuation of the effect of HSP90 inhibitor A. Heat map showing that genes were up-regulated by HSP90 inhibitor, but the upregulation was abolished by *HSF1* knockdown. *shNTC* or *shHSF1* transduced A375 cells were treated with or without Doxycycline for 3 days and were further treated with NVP-HSP990 100nM for 3h. Total RNA were collected and microarray was performed. B. Real-time PCR analysis of the expression of *DEDD2* gene in cells under HSP90 inhibitor treatment with or without *HSF1* knockdown. C. Western blotting analysis of protein expression in cells treated with HSP990 and *HSF1* shRNA. *shHSF1* transduced A375 cells were treated with or without Doxycycline for 3 days and were further treated with NVP-HSP990 100nM for 24h. D. Western blotting analysis of DEDD2 expression in cells with over-expression of *HSF1*. Inducible *HSF1* over-expressed cells were treated by Doxycycline for 3 day. The expression of HSF1 and DEDD2 were measured by western blotting. E. ChIP with HSF1 antibody in cells treated with NVP-HSP990. A375 cells were treated with HSP90 inhibitor for 1 hour. Chromatin was immunoprecipotated with anti-HSF1 antibody and amplified by quantitative real-time PCR using primers around HSE element of *DEDD2* gene promoter. F. Dose response of NVP-HSP990 in cancer cells with knockdown of either or both *DEDD* and *DEDD2*. A375 cells were treated with or without siRNA for 2 days, then followed by treatment of a serial dilutions of NVP-HSP990 for 3 days. Total RNA were also collected and real-time PCR was performed. Relative cell growth was measured by CellTiter-Glo and normalized to DMSO-treated cells.

Previous studies suggest that *HSP70/HSC70* knockdown has a combinational effect with HSP90 inhibitor, which might partially account for certain extend of the combinational effect of *HSF1* knockdown with HSP90 inhibitor [32]. To identify additional HSF1-target genes, we compared the gene profiles in A375 cells with or without *HSF1* knockdown treated or untreated with HSP90 inhibitor. Several genes are up-regulated by HSP90 inhibitor treatment and this upregulation are diminished by *HSF1* knockdown (Fig. 5A). Most of those genes belong to well-known HSF1-regulated cell stress pathway, such as: *HSPA1L, HSPA1A, HSPA6, HSPB1, HSPA4C and DNAJR1* (Fig. 5A). Among these top hits, apart from heat-shock pathway genes regulated by HSF1, BAG3 and DEDD2 are involved in cell death [33, 34]. Previously, BAG3 was identified as a HSF1 target gene and DEDD2 was defined as one of genes included in the molecular signature in response to the HSP90 inhibitor [35-37]. BAG3, a member of the Bcl-2-associated athanogen family, was reported as a mediator of a novel macroautophagy pathway that uses the specificity of HSP70 to misfolded proteins [33]. DEDD2 associates with DEDD and is involved in the regulation of nuclear events mediated by the extrinsic apoptosis pathway [37]. DEDD2 might be an important mediator for death receptors and target caspases to the nucleus [34]. However, the functional roles of BAG3 and DEDD2 in cancer cell response to HSP90 inhibition are not explored. Therefore, we decided to further validate whether *BAG3* and *DEDD2* may play a role in the combinational effect mediated by *HSF1* knockdown and HSP90 inhibition. Like *HSP70*, both *BAG3 and DEDD2* mRNA expression were increased upon HSP90 inhibition, which were abolished upon *HSF1* knockdown (Fig. 5B and Supplement Fig. S4). DEDD2 protein expression was not significantly regulated by HSP90 inhibition, but its basal level was decreased upon *HSF1* knockdown (Fig.5C). In comparison, HSP70 protein level was significantly increased by HSP90 inhibition, which was abolished by *HSF1* knockdown (Fig.5C). Furthermore, DEDD2 expression was elevated by over-expression of HSF1 (Fig. 5D). ChIP experiment also showed that HSF1 is bound to *DEDD2* promoter in response to HSP90 inhibition (Fig. 5E). These results suggest that *DEDD2* is a direct target gene of HSF1. *DEDD2* knockdown alone mildly enhances effect of HSP90 inhibitor (Fig. 5F). In comparison, BAG3 knockdown did not affect the activity of HSP90 inhibitor (Supplement Fig. S5). Taking consideration of the possible redundancy function of DEDD and DEDD2, knockdown of both *DEDD* and *DEDD2* was performed by using siRNA in the combinational experiment and a significant combinational effect with HSP90 inhibitor was observed (Fig. 5F). Thus, these results suggest that DEDD2 is involved in compensation mechanism of HSP90 inhibitor.

## DISCUSSION

Under heat shock condition, HSF1 monomers dissociate from HSP90, undergo trimerization, nuclear translocation and subsequently upregulate expression of heat shock proteins, including HSP70 and HSP27 [32]. The inhibition of HSP90 also increases HSF1 trimer stability and prolongs the heat shock response [38]. Previously, studies suggest that HSF1 transcriptional activity is upregulated by HSP90 inhibition, which may limit the efficacy of HSP90 inhibitors through the activation of heat-shock responsive genes including HSP27 and HSP70 [32]. However, the functional role of HSF1 itself in response to HSP90 inhibition is not fully appreciated. We found that knockdown of *HSF1* sensitizes cancer cells to HSP90 inhibitors in many different cancer lineages, including melanoma (A375, A2058), hepatocellular carcinoma (Hep3B, Huh7) and colon cancer (HCT116). More importantly, the combination of *HSF1* knockdown with HSP90 inhibition prevents tumor growth significantly in xenograft mouse model (stasis or regression). We also observed that the combined treatment with *HSF1* shRNA and HSP90 inhibitor leads to reduction of HSP90 inhibitor-induced HSP70 expression and p-ERK signal and induction of cleaved PARP.

Targeting HSF1 in combination with HSP90 inhibitor in HCC is particularly interesting because HSF1 expression level, both in mRNA and protein, is remarkable higher in primary HCC sample than normal hepatocytes. HSF1 knockout mice are viable and healthy, suggesting that inhibition of HSF1 might not be toxic to normal tissues [24]. Apart from combination with potential HSF1 inhibition, HSP90 inhibitor was also proposed to combine with mTOR inhibitor since blocking HSP90 may disrupt rapamycin-induced activation of alternative signaling pathways in HCCs and substantially improve the growth-inhibitory effects of mTOR inhibition *in vivo* [39]. HCC is an aggressive human cancer and current therapies are not very effective. HSP90 inhibitor in combination with HSF1 inhibitor or mTOR inhibitor may provide an additional therapeutic strategy for HCC.

The question is how to drug HSF1 as a transcription factor. A promising approach to target *HSF1* is to use therapeutic siRNA to knockdown *HSF1*. Although a significant progress has been made in developing siRNA therapy, there are still many hurdles to overcome [31]. Many efforts have also been put into drugging the HSF1 pathway. A couple of small compounds have been identified to have the ability to inhibit heat shock-induced upregulation of HSP and other HSF1 targets in cells, such as quercetin and KNK437[40]. In particular, the anti-malaria drug quinacrine (QC) was showed to prevent heat shock response in cancer cells and suppresses HSF1 induced HSP70 expression in a relatively selective manner [41]. In future, developing the potent and selective HSF1 pathway inhibitors might eventually be useful for treating human cancer in combination with HSP90 inhibitor or other agents.

We also compared the gene profile between melanoma cells treated with HSP90 inhibitor with or without *HSF1* knockdown. As expected, most genes regulated by HSF1 in response to HSP90 inhibitors are related to heat shock response. Some of them, such as *HSP70*, have been shown to have a combinational effect with HSP90 inhibitor previously [32]. *DEDD2* is up-regulated by HSP90 inhibition and this up-regulation is reversed by HSF1 knockdown. Interestingly, only DEDD2 is regulated by HSF1 while DEDD is not regulated by HSF1 under HSP90 inhibitor treatment (data not shown). Knockdown of both *DEDD2* and *DEDD*, but not either alone, enhances HSP90 inhibitor efficacy, suggesting the redundancy function of these proteins.

In this study, we identified that HSF1 transcriptional activities are induced by HSP90 inhibitors, which may provide a resistance mechanism through up-regulating a protective “heat shock” response and other transcriptional targets, such as *DEDD2*. However, a couple of questions still remain to be answered: 1) Are there any other transcriptional targets regulated by HSF1 required for HSF1-mediated resistance to HSP90 inhibitor in different settings? 2) Are there any co-regulators whose activities are required for HSF1-mediated resistance to HSP90 inhibitor? These questions will further prompt us to initiate large-scale screens to directly identify the HSF1-key-targets in different settings and HSF1-cofactors that are important for HSF1 transcriptional activities induced by HSP90 inhibitors in future. While a subset of HSF1-dependent-targets may play important roles in attenuating the efficacy of HSP90 inhibitors, the newly identified HSF1-target genes and/or HSF1-cofactors will not only help us to understand how HSF1 transcriptional function is regulated but may also reveal novel therapeutic targets in combination with HSP90 inhibitors.

## METHOD AND MATERIALS

### Cell Culture

A375, A2058, HCT116, Hep3B and Huh7 cells were obtained from American Type culture Collection. All cell lines were maintained in Dulbecco's Modification of Eagle's Medium, McCoy's 5a medium or advanced RPMI medium 1640 (Invitrogen) with 10% FBS (Invitrogen). Infected cell lines were maintained under 1 μg/mL of puromycin (MP Biomedicals) for selection.

### Cell Viability Assay

Cell viability at starting and ending day of compound treatment was determined by measuring cellular ATP content using the CellTiter-Glo luminescence assay (Promega). CellTiter-Glo reagent was added to each well and luminescence recorded on an Envision plate reader (Perkin Elmer). Luminescence values were used to calculate the inhibition of cell viability relative to DMSO-treated cells (0% inhibition) to calculate. Half maximal inhibitory concentration (IC50) and median lethal dosage (LD50) were further calculated.

### Pooled shRNA screening

pLKO.1 lentiviral plasmids encoding shRNAs targeting the kinases and apoptosis related genes were obtained and combined to generate a plasmid pool as well as control shRNAs designed not to target any gene. These plasmid pools were used to generate lentivirus-containing supernatants as described^23^. For screening, A375 cells were infected with the pooled virus so as to ensure that each cell contained only one viral integrant. Cells were selected for 3 days with 1μg/ml puromycin. After selection, 6×10^6^ cells were collected as Day0 sample. 6×10^6^ cells were further cultured and treated with or without NVP-AUY922 (10 or 20nM) for 14 days. Genomic DNA was isolated from cells by DNA extraction in Qiagen DNA blood and Tissue kit. To amplify the shRNAs encoded in the genomic DNA, PCR was performed for 33 cycles at an annealing temperature of 66 °C using 2-6 μg of genomic DNA, the primer pair indicated below, and DNA polymerase. Forward primer: CGGCGACCACCGAGATCttgggtagtttgcagttttaaaattatgt; reverseprimer1: CATACGAGATCTAGCAttctttcccctgcactgtaccccccaatcc; reverseprimer2: GCATACGAGATCGCATGttctttcccctgcactgtaccccccaatcc. After purification, the PCR products from each tumour were quantified by ethidium bromide staining after gel electrophoresis, pooled at equal proportions, and analysed by high-throughput sequencing (Illumina).

### Short Hairpin RNA Constructs

Control short hairpin RNA (shRNA), GGATAATGGTGATTGAGATGG, *HSF1* shRNA#1, GCAGGTTGTTCATAGTCAGAA, and *HSF1* shRNA#2, GCCCAAGTACTTCAAGCACAA, were cloned into the inducible pLKO-Tet-On puromycin vector as previously described.

### Lentivirus and Infection

Lentiviral supernatants were generated according to our previously established protocol. A total of 100 μL of lentivirus was used to infect 300,000 cancer cells in a six-well plate, in 8 μg/mL polybrene (Chemicon). Medium was replaced and after 24 h, cells were selected by puromycin (MP Biomedicals) and expanded. Induction of shRNA was obtained by addition of 100ng/mL Doxycycline (Clontech) to the medium.

### RNA Extraction and Quantitative Reverse Transcription-PCR

Total RNA was isolated using the RNeasyMini kit (Qiagen). ABI taqman gene expression assays include *HSP70* and *DEDD2*. VICMGB primers/probe sets (Applied Biosystems) were used in each reaction to coamplify the B2M transcripts. All experiments were performed in triplicate and normalize to B2M levels as indicated.

### Immunohistochemistry

A tissue microarray containing hepatocellular carcinoma samples with Hepatitis B-positive patients (50 cases, 2 cores per case) and two non-neoplastic liver samples (2 cores per sample) was purchased from AccuMax (A217). Primary antibodies were Ki67 antibody, Ki67_2 (Vector Laboratories Rabbit, VP-RM04), rabbit IgG isotype control (Southern Biotech, 0111-01) and HSF1 antibody (Cell Signaling Technology, Rabbit, 4356) were used. Secondary antibody incubation was done with either Ventana OmniMap or Ventana UltraMap prediluted HRP-conjugated multimer anti-rabbit secondary antibodies (Cat # 760-4315). Immunohistochemical staining was performed on the Ventana Discovery System. Images were captured using Aperio Scanscope.

### Chromatin Immunoprecipitation (ChIP) Assay

ChIP assay was carried out according to the manufacturer's protocol (chromatin immunoprecipitation assay kit, catalog no. 17-295, Upstate Biotechnology Inc, Lake Placid, NY). Immune complexes were prepared using anti-HSF1 (Cell Signaling, 4356) antibody. The supernatant of immunoprecipitation reaction carried out in the absence of antibody served as the total input DNA control. PCR was carried out with 10 μl of each sample using the following primers: *DEDD2* promoter, 5'-GAGTCACGGGCAGGAAGTAG-3' and 5'-ATTATTACGCCTGCGTCACC-3 '. This was followed by analysis on 2% agarose gels.

### Gene Profiling

RNA was isolated using the Qiagen RNeasy mini kit. Generation of labeled cDNA and hybridization to HG-U133 Plus2 arrays (Affymetrix) were performed as previously described [42]. DNA microarray results have been deposited at the Gene Expression Omnibus (GEO) under accession GSE44867.

### Western blotting

Western blotting was performed as follows: total tumor lysates were separated by SDS/PAGE and electrotransferredto nitrocellulose membrane (Invitrogen). Membraneswere blocked in PBS and 0.1% (vol/vol) Tween-20 (PBS-T) and 4% (wt/vol) nonfat dry milk (Bio-Rad) for 1 h on a shaker at room temperature. Primary antibodies were added to the blocking solution at 1:1,000 (HSF1; Cell signaling, 4356), 1:1,000 (HSP70; Cell signaling, 4876), 1:1,000(DEDD2 ; Abcam, ab104350), 1:1,000(p-ERK; Cell signaling, 4370), 1:1,000(ERK; Cell signaling, 4695), 1:1,000(HER2; Cell signaling, 4290), 1:1,000(BRAF; Cell signaling, 9433), 1:1,000(cleaved PARP; Cell signaling, 5625), and 1:10,000 (GAPDH; Cell Signaling Technology, 2118S) dilutions and incubated overnight and a rocker at 4 °C. Immunoblottings were washed three times, 5 min each with PBS-T, and secondary antibody was added at 1:10,000 dilution into PBS-T milk for 1 h on a shaker at room temperature. After several washes, enhanced chemiluminescence (ECL) reactions were performed according to manufacturer's recommendations (SuperSignal West Dura Extended Duration Substrate; Thermo Scientific).

### Tumor xenografts

Mice were maintained and handled in accordance with Novartis Biomedical Research Animal Care and Use Committee protocols and regulations. A375 and Hep3B cells engineered with Tet-inducible shRNA against HSF1 were cultured in DMEM and EMEM supplemented with 10% Tet-approved FBS. Mice (6–8 weeks old, n=8) were inoculated s.c. with 5 × 10^6^ A375 cells or 7 × 10^6^ Hep3B cells in the right dorsal axillary region. Tumor volume was measured by calipering in two dimensions and calculated as (length × width) / 2. Drug treatment started 6 days (A375) or 19 days (Hep3B) after implant when average tumor volume was around 200 mm^3^. Animals received vehicle (5% dextrose, 10 ml/kg, orally, qw), Doxycycline (25mg/kg, orally, qd) or NVP-HSP990 (10 mg/kg, orally, qw) for the duration of the study. At termination of the study, tumor tissues were excised and snap frozen in liquid nitrogen for immunoblotting analyses of biomarkers. Data were expressed as mean ± SEM, and differences were considered statistically significant at *P* < 0.05 by Student *t* test.

### Patient tumor sample analysis

RNAseq data for breast cancer (BRCA) and liver (LIHC) was retrieved from the TCGA data portal for both tumor and matched normal samples. The normalized RSEM values for each transcript were log2 transformed, and then Z score normalized for performing survival analysis.

### Statistics

Statistical analyses were performed by using Student *t* Test (*: *p*<0.05, **: *p*<0.01) (GraphPad Prism v5.01 software for Windows, GraphPad Software, San Diego, CA USA).

## SUPPLEMENTARY FIGURES AND TABLES




