# Supplementary material for: Targeting HSF1 sensitizes cancer cells to HSP90 inhibition
Source: Oncotarget. 2013 Apr 23;4(6):816–29. doi: 10.18632/oncotarget.991 (PMC3757240; doi:10.18632/oncotarget.991)
Supplement: Supplementary file 1 [file oncotarget-04-816-s001.pdf]

Targeting *HSF1* sensitizes cancer cells to HSP90 inhibition - Chen et al

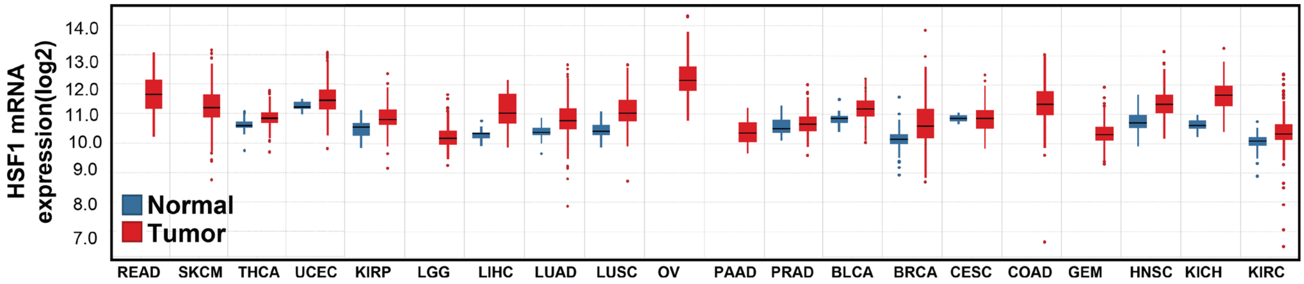

Supplementary Figure S1: The expression of HSF1 among different human cancers. Boxplot showed that comparisons of the RNAseq expression patterns for *HSF1* across TCGA tumor types with matched normal. Those human cancer types include Rectum adenocarcinoma (READ), Skin Cutaneous Melanoma (SKCM), Thyroid carcinoma (THCA), Uterine Corpus Endometrioid Carcinoma (UCEC), Kidney renal papillary cell carcinoma(KIRP), Brain Lower Grade Glioma(LGG), Liver hepatocellular carcinoma(LIHC), Lung adenocarcinoma(LUAD), Lung squamous cell carcinoma(LUSC), Ovarian serous cystadenocarcinoma(OV), Pancreatic adenocarcinoma(PAAD), Prostate adenocarcinoma(PRAD), Urothelial Carcinoma(BLCA), Breast invasive carcinoma(BRCA), Cervical squamous cell carcinoma and endocervial adenocarcinoma(CESC), Colon adenocarcinoma(COAD), Glioblastoma multiforme(GEM), Head and Neck squamous cell carcinoma(HNSC), Kidney Chromophobe(KICH) and Kidney renal clear cell carcinoma(KIRC).

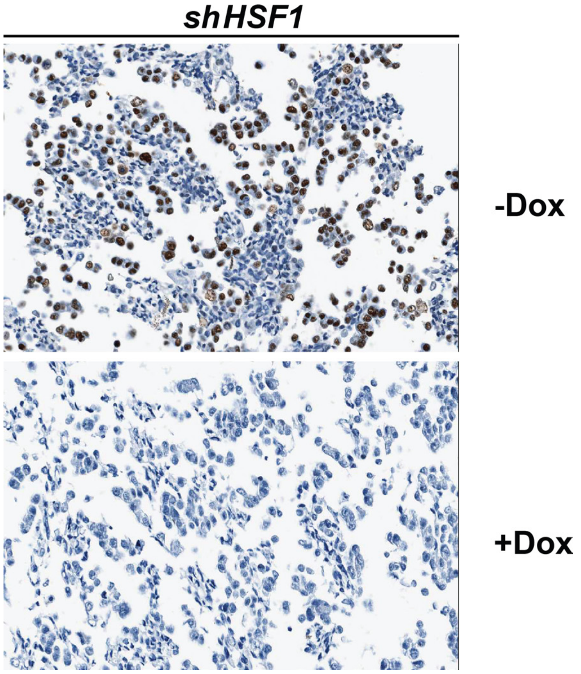

Supplementary Figure S2: Anti-HSF1 antibody showed a high specificity to HSF1 protein. Hep3B cell with inducible *HSF1* shRNA were treated with or without Doxycycline for 48h. The cell pellet was collected and the expression of HSF1 was measured by IHC.

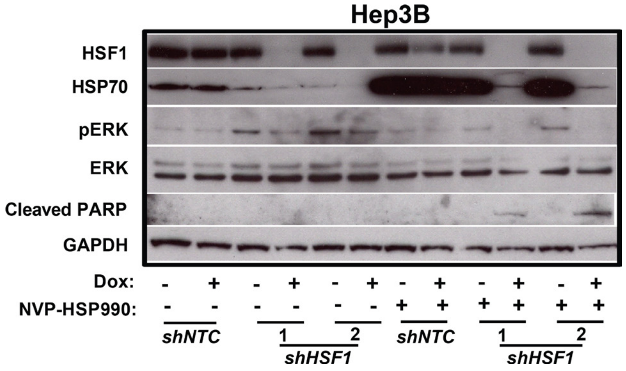

Supplementary Figure S3: The combination of HSF1 knockdown and HSP90 inhibitor leads to a decreased level of p-ERK and increased cleaved PARP in Hep3B cells. Supplementary Figure S4: Real-time PCR analysis showed that the expression of HSP70 and BAG3 were upregulated by HSP90 inhibitor and the upregulation was abolished by HSF1 knockdown

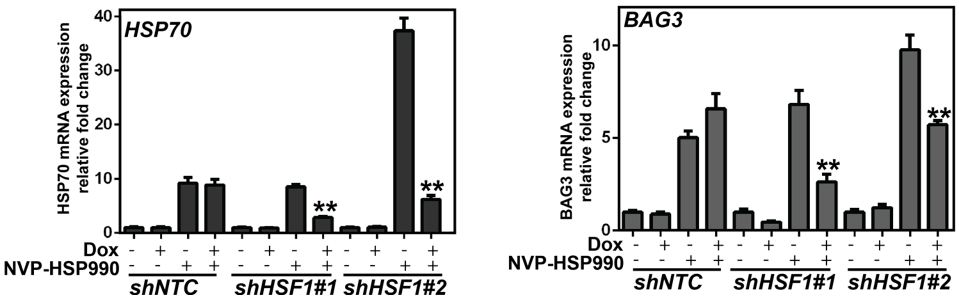

Supplementary Figure S4: Real-time PCR analysis showed that the expression of *HSP70* and *BAG3* were upregulated by HSP90 inhibitor and the upregulation was abolished by *HSF1* knockdown.

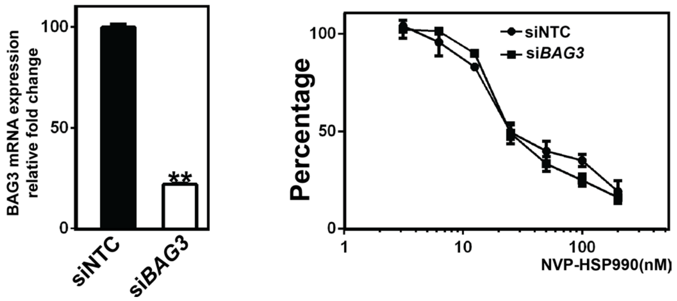

Supplementary Figure S5: Dose response of NVP-HSP990 in cancer cells with knockdown of BAG3. A375 cells were treated with or without siRNA for 2 days, then followed by treatment of a serial dilutions of NVP-HSP990 for 3 days. Total RNA were also collected and real-time PCR was performed. Relative cell growth was measured by CellTiter-Glo and normalized to DMSO-treated cells.
